# Altered patterns of gene duplication and differential gene gain and loss in fungal pathogens

**DOI:** 10.1186/1471-2164-9-147

**Published:** 2008-03-28

**Authors:** Amy J Powell, Gavin C Conant, Douglas E Brown, Ignazio Carbone, Ralph A Dean

**Affiliations:** 1 Department of Computational Systems Biology, Sandia National Laboratories, Albuquerque, NM, USA; 2 Smurfit Institute of Genetics, University of Dublin, Trinity College, Dublin, Ireland; 3 Department of Plant Pathology, Center for Integrated Fungal Research, North Carolina State University, Raleigh, NC, USA

## Abstract

**Background:**

Duplication, followed by fixation or random loss of novel genes, contributes to genome evolution. Particular outcomes of duplication events are possibly associated with pathogenic life histories in fungi. To date, differential gene gain and loss have not been studied at genomic scales in fungal pathogens, despite this phenomenon's known importance in virulence in bacteria and viruses.

**Results:**

To determine if patterns of gene duplication differed between pathogens and non-pathogens, we identified gene families across nine euascomycete and two basidiomycete species. Gene family size distributions were fit to power laws to compare gene duplication trends in pathogens *versus *non-pathogens. Fungal phytopathogens showed globally altered patterns of gene duplication, as indicated by differences in gene family size distribution. We also identified sixteen examples of gene family expansion and five instances of gene family contraction in pathogenic lineages. Expanded gene families included those predicted to be important in melanin biosynthesis, host cell wall degradation and transport functions. Contracted families included those encoding genes involved in toxin production, genes with oxidoreductase activity, as well as subunits of the vacuolar ATPase complex. Surveys of the functional distribution of gene duplicates indicated that pathogens show enrichment for gene duplicates associated with receptor and hydrolase activities, while euascomycete pathogens appeared to have not only these differences, but also significantly more duplicates associated with regulatory and carbohydrate binding functions.

**Conclusion:**

Differences in the overall levels of gene duplication in phytopathogenic species *versus *non-pathogenic relatives implicate gene inventory flux as an important virulence-associated process in fungi. We hypothesize that the observed patterns of gene duplicate enrichment, gene family expansion and contraction reflect adaptation within pathogenic life histories. These adaptations were likely shaped by ancient, as well as contemporary, intimate associations with monocot hosts.

## Background

Change in gene inventory in pathogenic genomes is an important evolutionary signal. Previous studies have documented the relationship between virulence and differential gene gain and/or loss in bacteria and viruses [1-8]. However, this phenomenon remains unexamined at a genomic scale in fungal pathogens.

Our exploration of patterns of differential gene gain and loss in pathogenic fungal genomes was prompted by two possibly related observations. First, gene counts in phytopathogenic euascomycete species and fungus-like plant parasites, such as species of *Phytophthora*, are often higher than those for the most closely related non-pathogenic genomes [9-17]. Second, some of the additional genes identified in these pathogens are predicted to have roles in secondary metabolism and managing encounters with hosts [10,13,18-21]. For instance, polyketide synthetases and non-ribosomal peptide synthetases are essential for toxin production, while G protein-coupled receptors and cytochrome P450s are critical for host perception and quenching infection-related oxidative stress [10,22-26].

The differential expansion of a gene family by duplication in a particular species is termed lineage-specific gene family expansion (LSE) [22,24,27,28]. Selection for virulence could induce LSE among particular gene families [10,11,13,18,22,24,29,30], as well as contraction among other families [31,32]. Size differences between the genomes of pathogenic and non-pathogenic species will depend on the relative rates of gene duplication, gene loss and horizontal transfer events. Two evolutionary trends that would result in larger genomes among pathogens are the consistent expansion of certain gene families [10,11,26], as well as pathogens' apparent affinity for gene acquisition through horizontal transfer [33-35]. However, it is also known that the number of genes in the genomes of opportunistic human fungal pathogens [36-40] appears to be reduced, as compared to non-pathogenic relatives, suggesting that gene loss may also be increased among some pathogens [41].

In the present study, we evaluated patterns of gene duplication in pathogens *versus *non-pathogens and in phylogenetically-informed paired species comparisons. We subsequently explored potential functional differences among duplicate genes in pathogens as compared to non-pathogens. In addition, we investigated trends of gene gain and loss in pathogenic fungal genomes.

## Results

### Altered patterns of gene duplication among diverse fungi

**Figure 1:**
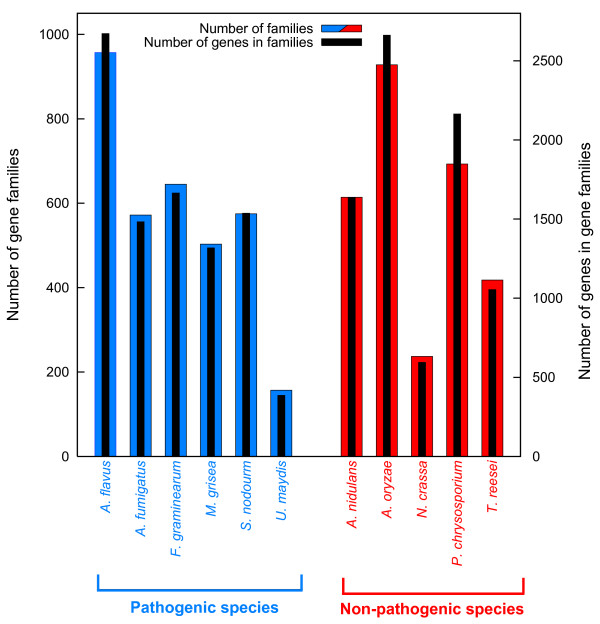
**Number and size of gene families larger than two for eleven fungal genomes**. Shown are the numbers of gene families with two or more members (red and blue bars) and the total numbers of genes in those gene families (black bars) across the sample of genomes studied here. Duplicate genes were identified by sequence similarity using GenomeHistory [82]. Duplicate genes were used to form homology-based single-linkage cluster gene families, using the graph-theoretic application GT Miner [89].

Gene duplicates identified by GenomeHistory in the eleven sequenced fungal genomes were grouped into gene families *via *single linkage clustering procedures (Methods). Figure 1 and Table 1 give the number of gene families of size two or greater that met all minimum threshold criteria, as well as the total number genes in gene families for each species. *U. maydis*, a basally branching hemibiotrophic pathogenic basidiomycete lineage, had the fewest gene families and the least number of genes in gene families, while the opportunistic euascomycete pathogen *A. flavus *possessed the greatest number of gene families, and also the largest number of genes in gene families.

**Table 1:** Summary of life history attributes for the genomes studied

**Species**	**Genes in genome**	**Gene families**	**Genes in families**	**RIP**^1^	**Classification**	**Life style**	**Primary reproductive mode**
*Aspergillus flavus*	12197	957	2672	yes	euascomycete	pathogen	asexual^2^
*Aspergillus oryzae*	12079	928	2662	yes	euascomycete	non-pathogen	asexual^2^
*Aspergillus fumigatus*	9926	572	1483	yes	euascomycete	pathogen	asexual^2^
*Aspergillus nidulans*	10701	614	1637	yes	euascomycete	non-pathogen	homothallic
*Stagonospora nodorum*	16597	575	1537	yes	euascomycete	pathogen	heterothallic
*Magnaporthe grisea*	12841	503	1318	yes	euascomycete	pathogen	asexual^2^
*Neurospora crassa*	10620	237	594	yes	euascomycete	non-pathogen	heterothallic
*Fusarium graminearum*	11640	645	1664	yes	euascomycete	pathogen	homothallic
*Trichoderma reesei*	9997	418	1054	yes	euascomycete	non-pathogen	heterothallic
*Ustilago maydis*	6522	157	386	no	basidiomycete	pathogen	heterothallic
*Phanerochaete chrysosporium*	10048	693	2164	no	basidiomycete	non-pathogen	heterothallic

^1^The stringency and efficiency of RIP-like processes varies among euascomycete genomes.

^2^Asexual propagation is the most frequently observed reproductive mode in field settings. However, asexual lineages often either have the potential for sexual reproduction, as indicated by the presence of different mating types in populations, and/or phylogenetic evidence for recombination and cryptic speciation.

We initially predicted greater proportions of duplicated genes would become fixed in pathogenic lineages as a result of increased preservation of duplicated genes by natural selection and/or higher rates of duplication. In this coevolutionary arms race scenario, continually evolving host resistance would give rise to constant selective pressure for the preservation of duplications of genes relevant to virulence [20,42,43]. We thus compared the distributions of gene family sizes in pathogens *versus *non-pathogens. The distribution of gene family sizes in a genome is thought to follow a power law distribution [44-47], and we therefore modeled the pooled set of pathogenic gene families as following this distribution. Similarly, we allowed the pooled set of non-pathogenic gene families to follow a power law distribution, with a potentially different value of the power law coefficient, *a *(which describes the frequency of gene families of each size; see Methods). We then applied a likelihood ratio test to examine the null hypothesis that the value of *a *was the same in pathogens as in non-pathogens.

Although we can reject the hypothesis of equal values of *a *(*P *< 10^-8^), surprisingly, in view of our initial prediction, we find that non-pathogenic fungi in fact have slightly larger gene families than do the pathogens (*a *= 4.14 *verses a *= 4.29 for pathogens). Interestingly, when the two basidiomycete species (*P. chyrosporium *and *U. maydis*) are excluded from the analysis, no overall significant differences in gene family size distribution are evident (*P *= 0.14). We also compared lineages that were primary pathogens (*U. maydi*s, *M. grisea*, *F. graminearum *and *S. nodorum*) to their non-pathogenic relatives (*P. chrysosporium*, *A. nidulans*, *N. crassa *and *T. reesei*), finding again that the non-pathogens have larger gene families (*a *= 4.57 and 4.26, respectively, *P *< 10^-17^). Again, excluding the basidiomycete species results in no significant difference being found (*P *= 0.28). Finally, we examined only the *Aspergillus *species, comparing the opportunistic pathogens *A. flavus *and *A. fumigatus *to *A. nidulans *and *A. oryzae*. No significant differences in gene family sizes are evident in the comparison between opportunistic pathogens and their non-pathogenic relatives (*P *= 0.15).

**Figure 2:**
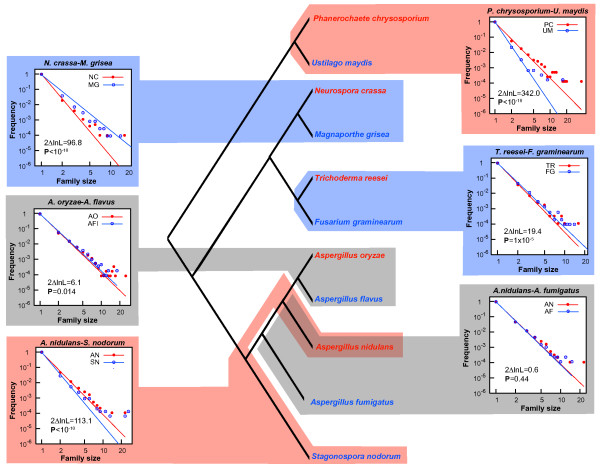
**Independent phylogenetic contrasts for pathogens and their closest non-pathogenic relatives**. Recent phylogenomics studies support relationships presented here [48, 49]. The distribution of gene family sizes in each genome is assumed to follow a power law, and the data fit to this distribution by maximum likelihood. Family size *versus *frequency data shown here are plotted on log-log-scales. Likelihood ratio tests were used to determine if pathogens (blue text) had larger gene families (blue shading), smaller gene families (red shading) or no significant difference in the distribution of gene family sizes distribution (grey shading), as compared to their closest non-pathogenic (red text) relative. *P*-values indicate the significance of these tests (with the null hypothesis that the power law coefficient, *a*, is the same for the pathogenic and the non-pathogenic species in each paired comparison). Values for the differences in the log likelihoods (*i.e*., 2ΔlnL) used to infer *P*-values are also given.

The above approach is potentially flawed, because the species in question do not represent independent realizations of the same general stochastic process. Rather, the genomes are related by the phylogeny shown in Figure 2[48,49]. To control for this common ancestry, we performed phylogenetically independent contrasts for gene family size distributions for the six species pairs indicated in Figure 2, applying the same likelihood ratio test described above. We find evidence for larger gene families in phytopathogenic species for two paired-species comparisons (*N. crassa versus M. grisea *and *T. reesei versus F. graminearum*; *P *≤ 10^-5^), while the other comparisons either showed either the opposite trend (*A. nidulans versus S. nodorum *and *P. chrysosporium versus U. maydis*; *P *< 10^-9^) or no significant differences (*A. flavus versus A. oryzae *and *A. fumigatus versus A. nidulans*; P > 0.01). In all cases, we used a significance threshold of α = 0.008, which reflects application of a Bonferroni correction for 6 hypothesis tests. Note that a maximum likelihood fit of another potential distribution describing the probability of observing a gene family of size *x *in a genome, the exponential distribution, visually provides a rather poorer explanation of these data (**see **Additional File 1).

### Functional distribution of gene duplicates in pathogens versus non-pathogens

**Figure 3:**
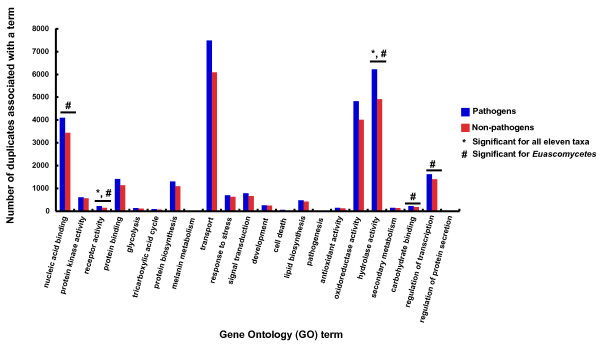
**Functional distribution of gene duplicates in pathogenic and non-pathogenic fungal lineages**. The distribution of gene duplicates across a sample of 22 Gene Ontology (GO) terms is compared for pathogenic (blue bars) and non-pathogenic (red bars) fungal lineages. When all eleven taxa were considered, we observed significantly higher proportions of gene duplicates associated with the terms "hydrolase activity" and "receptor activity" in pathogens (*); survey of the euascomycetes indicated that gene duplicates associated with the "hydrolase activity," "carbohydrate binding," "nucleic acid binding," "regulation of transcription," and "receptor activity" terms, respectively, were enriched in pathogenic species (**#**).

To elucidate potential functional differences in duplicated genes in pathogenic *versus *non-pathogenic genomes, we compared the distribution of GO terms between the two groups. We initially selected twenty-two different GO terms (Figure 3) representing functional categories that are relevant to fungal pathogenesis, as well as others related to basal metabolic processes. We compared the proportions of gene duplicates associated with a GO term for pathogens and non-pathogens.

When all eleven taxa were considered, two GO terms differed significantly in the number of gene duplicates observed for pathogens *versus *non-pathogens, after allowing for a 20% false discovery rate (FDR, see Methods). The "receptor activity" and "hydrolase activity" terms showed significantly greater numbers of gene duplicates in pathogenic species than in non-pathogenic lineages. When the same analysis was repeated for the nine euascomycete genomes, we identified three additional functional categories where pathogenic species had a greater than expected number of gene duplicates: "nucleic acid binding," "carbohydrate binding" and "regulation of transcription."

We also compared the number of gene duplicates associated with terms in the Generic GO Slim Ontology for pathogens and non-pathogens. This survey revealed no significant distinctions among pathogens *versus *non-pathogens for all taxa. When only euascomycete species were considered, we found that gene duplicates associated with following six terms were over represented among pathogens, again controlling for an FDR of 20%: "hydrolase activity," "extracellular region," "carbohydrate metabolism," "nucleobase, nucleoside, nucleotide and nucleic acid metabolism," "carbohydrate binding" and "catalytic activity" (Table 2).

**Table 2:** GO terms that are over represented in euascomycete pathogens

**Generic GO Slim Ontology (GO) Term**	**Number of gene duplicates in euascomycete pathogens**	**Number of gene duplicates in euascomycete non-pathogens**	**Initial significance value from chi-square test‡**	**Corrected significance value§**
hydrolase activity	5828	4916	1.60E-04	2.00E-03
carbohydrate metabolism	1724	1416	2.40E-03	5.90E-03
carbohydrate binding	229	177	6.50E-03	9.80E-03
extracellular region	314	227	1.94E-03	3.90E-03
nucleobase, nucleoside, nucleotide and nucleic acid metabolism	4131	3823	3.63E-03	7.80E-03
catalytic activity	16645	14696	1.13E-02	1.18E-02

**‡ **Significance values in this column are *P*-values obtained in chi-square tests

**§**Significance values presented in this column are corrected for Type 1 error (see Methods for FDR correction)

### Functional distribution of gene duplicates in species pairs

For the four species pairs that showed global differences in the magnitude of gene duplication (Figure 2), we surveyed the functional distribution of gene duplicates using the Generic GO Slim Ontology.

The rice blast fungus, *M. grisea*, showed higher average gene family size than its non-pathogenic relative *N. crassa *(Figure 2). Correspondingly, we found four GO terms that are overrepresented in pathogenic *M. grisea*, as compared to exclusively saprophytic *N. crassa *(Table 3). Further, while pathogenic *F. graminearum *had larger gene families than did non-pathogenic *T. reesei *(Figure 2), we found five overrepresented GO terms in *F. graminearum *and an equal number of overrepresented terms in *T. reesei *(Tables 3 and 4). When we compared the species pairs where the non-pathogenic taxon possessed more gene duplicates globally, (*A. nidulans versus S. nodorum *and *P. chrysosporium versus U. maydis*), we found significantly more gene duplicates associated with a total of eighteen particular GO terms in the *A. nidulans-S. nodorum *test pair, and fourteen terms in *P. chrysosporium *(*versus U. maydis*), respectively (Figure 2; Table 3). Significant differences in paired species comparisons were determined after applying corrections for multiple tests, as above.

**Table 3:** GO terms that are over represented in one member of a species pair

**Species pair**	**Generic GO Slim Ontology (GO) Term**	**Species with over-represented GO terms**
*M. grisea*/*N. crassa*	electron transport **‡**	*M. grisea*
*M. grisea*/*N. crassa*	transport	
*M. grisea*/*N. crassa*	generation of precursor metabolites and energy **‡**	
*M. grisea*/*N. crassa*	catalytic activity **‡**	

*F. graminearum*/*T. reesei*	transport	*F. graminearum*
*F. graminearum*/*T. reesei*	transporter activity	
*F. graminearum*/*T. reesei*	electron transport **‡**	
*F. graminearum*/*T. reesei*	generation of precursor metabolites and energy **‡**	
*F. graminearum*/*T. reesei*	catalytic activity **‡**	

*F. graminearum*/*T. reesei*	cytoplasm	*T. reesei*
*F. graminearum*/*T. reesei*	lysosome	
*F. graminearum*/*T. reesei*	Intracellular	
*F. graminearum*/*T. reesei*	cellular component organization and biogenesis	
*F. graminearum*/*T. reesei*	organelle	

*A. nidulans*/*S. nodorum*	DNA Binding	*A. nidulans*
*A. nidulans*/*S. nodorum*	transcription regulator activity	
*A. nidulans*/*S. nodorum*	transcription	
*A. nidulans*/*S. nodorum*	regulation of biological process	
*A. nidulans*/*S. nodorum*	nucleus	
*A. nidulans*/*S. nodorum*	nucleobase nucleoside nucleotide and nucleic acid	
*A. nidulans*/*S. nodorum*	metabolic process	
*A. nidulans*/*S. nodorum*	nucleic acid binding	
*A. nidulans*/*S. nodorum*	cytoplasm	
*A. nidulans*/*S. nodorum*	intracellular	
*A. nidulans*/*S. nodorum*	catalytic activity **‡**	
*A. nidulans*/*S. nodorum*	electron transport **‡**	
*A. nidulans*/*S. nodorum*	generation of precursor metabolites and energy **‡**	
*A. nidulans*/*S. nodorum*	organelle	

*A. nidulans*/*S. nodorum*	peptidase activity	*S. nodorum*
*A. nidulans*/*S. nodorum*	catabolic process	
*A. nidulans*/*S. nodorum*	antioxidant activity	
*A. nidulans*/*S. nodorum*	extracellular region	

*P. chrysosporium*/*U. maydis*	electron transport **‡**	*P. chrysosporium*
*P. chrysosporium*/*U. maydis*	carbohydrate binding	
*P. chrysosporium*/*U. maydis*	extracellular region	
*P. chrysosporium*/*U. maydis*	response to abiotic stimulus	
*P. chrysosporium*/*U. maydis*	generation of precursor metabolites and energy **‡**	
*P. chrysosporium*/*U. maydis*	nucleic acid binding	
*P. chrysosporium*/*U. maydis*	nucleotide binding	
*P. chrysosporium*/*U. maydis*	catalytic activity **‡**	
*P. chrysosporium*/*U. maydis*	protein complex	
*P. chrysosporium*/*U. maydis*	peptidase activity	
*P. chrysosporium*/*U. maydis*	multicellular organismal development	
*P. chrysosporium*/*U. maydis*	amino acid and derivative metabolic process	
*P. chrysosporium*/*U. maydis*	reproduction	
*P. chrysosporium*/*U. maydis*	catabolic process	

**‡ **Enrichment in pathogens and non-pathogens across four different pairwise comparisons

**Table 4:** GO terms that are over represented or under represented in pathogenic *F. graminearum versus *non-pathogenic *T. reesei*

**Generic GO Slim Ontology (GO) Term**	**Number of gene duplicates in pathogenic *F. graminearum***	**Number of gene duplicates in non-pathogenic *T. reesei***	**Initial significance value from chi-square test‡**	**Corrected significance value§**
catalytic activity	3214	2784	1.69E-02	1.69E-01
transport	1398	1086	2.29E-05	2.29E-03
transporter activity	741	550	1.24E-04	6.22E-03
electron transport	466	352	5.16E-03	1.65E-01
generation of precursor metabolites and energy	559	436	9.66E-03	1.65E-01
lysosome**†**	1	8	1.38E-02	1.65E-01
cytoplasm**†**	729	762	1.19E-02	1.65E-01
organelle**†**	1136	1166	6.95E-03	1.65E-01
intracellular**†**	1425	1432	1.46E-02	1.65E-01
cell organization and biogenesis**†**	280	314	1.48E-02	1.65E-01

**† **Denotes duplicate enrichment for the term in non-pathogenic *T. reesei*

**‡ **Significance values in this column are *P*-values obtained in chi-square tests

**§**Significance values presented in this column are corrected for Type 1 error (see Methods for FDR correction)

### Expansion or contraction of gene families in pathogens

To determine whether a given gene family showed significant expansion or contraction, we employed a binomial test (see Methods). Sixteen gene families show significant expansion in pathogens, while five gene families appear to be contracted. Functional identities, as well as gene family sizes are presented for all significantly expanded or contracted gene families in Additional files 2 and 3.

Some significantly expanded gene families of particular interest are those with predicted hydrolytic, transporter or oxidioreductase activities, as well as those involved with carbohydrate metabolism (see Additional file 2). Examples of expanded gene families in the hydrolytic functional class included those with predicted chitin deactylase, cutinase, amino peptidase and feruloyl esterase activities, among others. Specific examples of expanded gene families that belong to the oxidoreductase functional class included those with predicted galactose oxidase and tyrosinase activities. The transporter functional class of expanded gene families included instances of sugar porters, malic acid transporters, neutral amino acid permeases and L-fucose permeases, among others.

For gene families that were deemed significantly contracted, the main functional categories, as indicated by GO and GenBank annotations, also include hydrolases, oxidases and transporters. However, the specific biological roles of particular gene families that showed contraction in pathogens differ from those that showed significant expansion (see Additional files 2 and 3). For instance, in the oxidoreductase functional class, homologues of *ordA *(*O*-methylsterigmatocystin reductase) appear to be depleted in pathogens. In aflatoxin-producing species of *Aspergillus*, this gene catalyzes the last reaction in the biosynthesis of this secondary metabolite [31,50,51]. Other interesting examples include gene families that were predicted to be components of the vaculolar ATPase complex, in addition to those encoding glucose oxidase precursors (see Additional file 3).

### Expanded gene families in species that have more duplicated genes

For the four cases where phylogenetic contrast analyses indicated significant differences in gene family sizes in paired species comparisons (Figure 2), gene family expansion was also examined using an approach similar to that above (see Methods).

Five gene families are significantly expanded in *M. grisea*, relative to non-pathogenic *N. crassa*. These gene familiesinclude those predicted to have oxidoreductase, transporter, peroxidase and melanin biosynthesis functions (see Additional file 3). Four gene families are significantly expanded in *F. graminearum versus T. reesei*, including those predicted to have transporter, endoglucanase and methyl transferase activities (see Additional file 4). Interestingly, the significantly expanded gene family with methyl transferase functional attributes has substantial homology to *LaeA*, a gene that may play a global regulatory role in secondary metabolism in *Aspergillus *species [51,52]. There are two significantly expanded gene families in *A. nidulans*, relative to *S. nodorum *(see Additional file 4). One family is predicted to have phosphorylase activity, while the other encodes genes with ATP-binding cassette (ABC) transporter function (see Additional file 4). *P. chrysosporium *has eight significantly expanded gene families, relative to pathogenic *U. maydis*. These eight families include genes with predicted oxidoreductase and peptidase functions, as well as genes with roles in carbohydrate metabolism (see Additional file 4).

## Discussion

### Change in gene inventory and fungal genome evolution

Differential gene gain and loss clearly play definitive roles in both degree of virulence, and in determining host range [2,5-8,34,53,54]. Although there are numerous examples of gene family expansion in pathogenic lineages of fungi [10,22,24,26,29,31,38,53], to date, gene duplication trends across whole genomes have not been analyzed.

Our analysis sought to understand whether the sum of gene family expansions have given rise to an overall increase in numbers of gene duplicates in the genomes of pathogenic fungi. We observed no such trend when all pathogenic species were compared to the non-pathogens, nor when we performed phylogenetically-informed paired species comparisons. Instead, the paired-species comparisons showed no clear association between gene family size distributions and pathogenicity. An example of this lack of association is apparent in the *Aspergillus *species comparisons, where we found no significant differences in gene family size distributions, a result that derives from both the opportunistic nature of the pathogens, as well as the close phylogenetic relationships of the species in question [16,17,37,40]. Another potential explanation for similarity among the complement of gene duplicates in genomes of the Aspergilli is the recent suggestion that host animals, and humans in particular, do not generate the sort of co-evolutionary arms-race characteristic of the plant hosts of the other pathogenic species considered [55].

A confounding factor for our analyses is that gene duplication's ability to drive biological innovation is diminished in some fungi by the process of repeat-induced point mutation [9,56-59]. RIP is a pre-meiotic homology-based mechanism that introduces characteristic point mutations into sequences present in multiple copies in a genome, and may have evolved to limit the deleterious effects of mobile genetic elements [56]. All euascomycete genomes surveyed here likely possess some degree of RIP [9-12,15,36,56-58]. However, the severity and efficiency of the process varies, with *N. crassa *possibly possessing the most stringent form [56]. We note that *N. crassa *shows the smallest average gene family size among the species studied, as would be expected, given RIP's severe pruning of duplicates in this organism. There is no evidence for RIP in either of the basidiomycete genomes examined in this study, although it has recently been documented in anther smut [18,60,61]. Some of the differences illustrated in Figure 2 (particularly the differences between *M. grisea *and *N. crassa*) are possibly due to variation in RIP stringency rather than pathogenicity.

Although quite variable, the approximate divergence times among the six pathogenic and five non-pathogenic species examined are reasonable for large-scale comparative studies of gene duplication in euascomycete and basidiomycete lineages [36,62,63].

### Functional patterns of duplicate gene enrichment in fungal genomes

Enrichment of gene duplicates over particular functional categories in the set of twenty-two GO terms, as well as those in the Generic GO Slim Ontology appear consistent with requirements of pathogenic lifestyles [26,64-67]. These results parallel those of previous studies, which have demonstrated that fungal pathogenesis is associated with increased catalytic potential among enzymes such as hydrolases, relatively larger repertoires of receptors, and the expansion of secreted proteins, as well as carbohydrate recognition and binding gene families [10,11,18,25,26,29,67].

For example, we found an increased number of duplicates associated with transport functions in *F. graminearum*, the causal agent of head blight in cereals, as compared to non-pathogenic *T. reesei *(Tables 3 and 4), a result consistent with the known relative deficit of carbohydrate catalysis genes in *T. reesei *[12].

Some of the differences observed between non-pathogenic *P. chrysosporium *and the hemibiotrophic corn smut fungus *U. maydis *appear relevant to the markedly divergent ecology and development these basidiomycete species [18,60]. The over-represented terms "carbohydrate binding," "extracellular region," "response to abiotic stimulus," "multicellular organismal development," "peptidase activity," as well as "amino acid and derivative metabolic process" are associated with the suite of traits that make the white rot fungus, *P. chrysosporium*, amenable to industrial applications, such as lignocellulose and organopollutant degradation (Table 3). Moreover, the observed enrichment of gene duplicates dedicated to particular developmental programs, such as muticellularity, is also consistent with the differences in morphological complexity between these two organisms in their fruiting body structures; *U. maydis *produces no fruiting body, *per se*, only teliospore-filled tumors on a host, whereas *P. chrysosporium *possesses a resupinate fleshy fruiting body. That *U. maydis *showed no enrichment for gene duplicates for any GO term was consistent with recent analyses of this genome, which indicated relatively few duplicates [18].

When phylogenetic relationships are not considered, a cohort of GO terms that is common to pathogens and non-pathogens in all four paired-species comparisons becomes evident (see Table 3). These three terms are: electron transport, generation of precursor metabolites and energy, as well as catalytic activity. Biological explanations for this observation include two possibilities that are not mutually exclusive: first, that genes associated with these three terms are not peculiar to pathogenesis, and/or secondly, that genes associated with these terms possess inherently greater plasticity in copy number in fungal genomes. Given numerous accounts of duplication in pathogenic and non-pathogenic fungi, copy number plasticity in certain functional categories would seem the more plausible explanation.

Our results, both from comparisons across all taxa, as well as paired species comparisons, suggest an association between organisms' lytic potential ("hydrolytic activity") and receptor ("receptor activity") repertoires and pathogenesis (Figure 3; Tables 2 and 3). Moreover, even when a pathogen had fewer gene duplicates overall (*A. nidulans versus S. nodorum*), enrichment of terms associated with lytic activities ("peptidase activity," "catabolic process"), management of oxygen toxicity ("antioxidant activity"), as well as terms possibly relevant to protein secretion ("extracellular region") were still evident in the pathogenic lineage (Table 3).

### Gene family expansion or contraction in pathogens

Gene families that were expanded in pathogens largely fall into two functional categories: those encoding lytic enzymes and those encoding putative transporters (see Additional file 2). Lytic enzymes, such as feruloyl esterases, cutinases, aminopeptidases and endoglucanases are known to be significant in successful plant parasitism interactions, as such genes have demonstrated roles in plant cell wall decomposition [26,30,64]. Serine proteinases and the regulatory P domain of the subtilisin-like proprotein convertases have been implicated in mutualistic, as well as pathogenic, interactions with grasses [26,68,69]. Moreover, the importance of fungal chitin deactylases in entomopathology has been empirically demonstrated [70]. Expanded families that were predicted to have diverse transporter functions included genes that encode L-fucose and neutral amino acid permeases, as well as MFS, malic acid and sugar transporters, respectively (see Additional file 2). L-fucose permeases have not only been implicated in galactose transport and sphigolipid metabolism, but also in development of resistant sclerotia in *A. flavus*, while neutral amino acid permeases and MFS transporters are possibly involved in efflux of non-ribosomal peptides and other secondary metabolites required for virulence [71,72]. Permeases may also be critical for the initial assault upon a host, as well as assimilation of host-derived carbohydrates [73,74]. The remaining expanded families included those encoding tyrosinases, galatose oxidase precursors and transmembrane receptors, all of which have been implicated in pathogenic life styles [26,66,75]. Tyrosinases catalyze initial steps in melanin biosynthesis [76]. Melanization in fungi is frequently, if not always, essential for virulence [26,65,66]. It is notable that another gene in the melanin synthesis pathway was also found to be over duplicated in *M. grisea*, relative to non-pathogenic *N. crassa *[77]. Galactose oxidation yields peroxide [78], a reactive oxygen species known to be produced by fungal pathogens during infection [19,79]. Recent work in *M. grisea *described a phalanx of receptors that are linked to pathogenesis and host perception [29].

The five families that showed gene losses in pathogens included those with predicted roles in secondary metabolism (see Additional file 3). One intriguing case is that of *O*-methylsterigmatocystin oxidoreductase, which catalyzes the last step in aflatoxin biosynthesis. While only a few species of *Aspergillus *are known to produce aflatoxin, numerous euascomycete and basidiomycete lineages possess homologues of this gene, as well as others in the aflatoxin biosynthesis pathway [32,51]. The wide phylogenetic distribution of these genes suggests not only that that these genes are ancient, but also that they are possibly important in secondary metabolism. We speculate that contraction of this family in pathogenic lineages may be the result of random gene deletions after relaxation of selective pressure that maintains production of this toxin [31,32,80,81]. Recent genomic and population studies that focused on the aflatoxin biosynthesis cluster in species of *Aspergillus *would seem to support this conjecture, as *ordA*, an *O*-methylsterigmatocystin oxidoreductase, appears to have undergone more extensive loss following duplication than any other gene in the cluster [22,31].

### Gene family expansion in species pairs

Gene families that were expanded in two paired-species comparisons also had either expected or previously demonstrated roles in virulence, host-pathogen interaction or chemical defense [22,26,66,67]. For example, homologues of *laeA*, a methyltransferase, are over duplicated in *F. graminearum*. This gene appears to be a global regulator of secondary metabolism in species of *Aspergillus *[51,52]. It is interesting to speculate whether an expanded repertoire of upstream regulators of secondary metabolism in *F. graminearum *could reflect either greater numbers of target genes or refined regulatory control of secondary metabolic networks in this primary pathogen (see Additional file 4). In the second instance where a phytopathogen had overall greater levels of gene duplication, five gene families were expanded. Gene families encoding diverse cytochrome P450s, MFS transporters, as well as those predicted to have trihydroxynaphthalene reductase activity were expanded in *M. grisea*, as compared to exclusively saprophytic *N. crassa *(Figure 2; see Additional file 4).

The white rot fungus *P. chrysosporium *had eight gene families that were significantly expanded, relative to basal basidiomycete *U. maydis *(see Additional file 4). These eight expanded families encode genes that have diverse oxidoreductase activities, protease and endo-1, 3 (4)-beta-glucanase functions, consistent with genes previously reported to be present in high copy numbers in this genome [60]. A large complement of such genes in this organism's genome is likely a reflection of its saprotropic wood-decay ecology.

## Conclusion

Here, we present results on patterns of gene duplication and differential gene gain and loss in pathogenic fungal genomes. The scope of this study captures at least of one billion years of fungal evolution [62]. No general relationship between pathogenicity and the magnitude of gene duplication was evident, but differences in duplicate gene retention among certain functional classes were consistent with known and predicted requirements of virulent lifestyles [26,66,67,75]. Gene family expansion in species with overall higher levels of duplication also showed functional trends that could be reconciled with organismal life histories [9-12,15,18,36,37,55,60,66,75]. The observed differences in overall levels of duplication between phytopathogenic lineages and their non-pathogenic relatives implicate gene inventory flux as an important virulence-associated process in fungi.

## Methods

### Identifying gene duplicates within and among fully sequenced genomes

Gene duplicates in nine euascomycte genomes, and two basidiomycete genomes (Table 1) were identified using a customized version of GenomeHistory (GH), which was parallelized for implementation in a high performance computing environment [82]. Each of these genomes is publicly available [83-86]. Our GH analyses required that candidate duplicate genes have at least 40% amino acid identity and a BLAST-based significance threshold of E ≤ 10^-8^.

### Forming sequence homology-based single linkage clustering gene families

Candidate gene duplicates obtained in the above GH analyses that had BLAST hits with query and subject coverage of at least 40%, a minimum percent identity of 25% and significance threshold of E ≤ ^-3 ^to Repbase V11.05 sequences were excluded from further analysis [87,88]. Homology-based single linkage clustering (SLC) gene families were formed using standard graph theoretic approaches and network graphing algorithms within the GT Miner software package [89-91]. Our approach required that a gene family have at least two members. Candidate gene duplicate pairs obtained in the above GH analyses were also filtered using minimum coverage thresholds, where query and subject sequences were required to show at least 70% coverage, prior to forming single-linkage clustering gene families. Single linkage gene families were computed across all eleven genomes (Figure 1).

### Distribution of gene family sizes in pathogenic species verses non-pathogenic species

To assess the relative likelihood of observing gene families of a given size in pathogenic and non-pathogenic species, we compared the distribution of gene family sizes across eleven genomes. This comparison assumes that the probability of observing a gene family with *n *members is given by:

$$P=\frac{n^{-a}}{\sum_{i=1}^{\infty }i^{-a}}$$

For this distribution, the value of *a *can be thought of as the "rate of decay" of gene family sizes: large values of *a *indicate proportionally more small gene families. Thus, we can thus compare values of *a *between genomes to test for genome-scale differences in duplication propensities. Given the observed distribution of family sizes in each genome, we used numerical optimization to find the value of *a *that gives the maximum likelihood of observing these gene family sizes [92]. We first compared the overall distribution of gene families in pathogens to that in the non-pathogens. We separately fit these two sets of gene families to the above distribution by maximum likelihood and retained the ln-likelihood of each dataset (lnL_P and lnL_NP). We then created a pooled dataset containing all gene families from both the pathogens and non-pathogens and calculated the likelihood of that pooled dataset (lnL_A). This pooled analysis is a special case of the previous analyses, where the pathogens and non-pathogens are required to have the same value of *a *(*i.e*., required to have the same gene family size distributions). To test the hypothesis of equal values of *a *between the datasets, we compared twice the difference in log-likelihood between the pooled model and the sum of the other two likelihoods (*i.e*., 2Δln *L *= 2((ln *L*_*P *+ ln *L*_*NP*) - ln *L*_*A*)). Under the null hypothesis of no difference between the pathogens and non-pathogens, 2ΔlnL will follow a chi-square distribution, with one degree of freedom [93]. Cases where the *P*-values from these tests were small (<0.05) indicated significant differences in duplication propensities in the genomes being compared. We employed an identical procedure for testing for significant differences among individual pairs of genomes.

### GeneOntology-based functional annotation

Gene Ontology (GO) terms were associated with genes in gene families using the 2006 version of the GO Consortium's database [94,95]. All genes in gene families were queried against proteins extracted from the GO database using BLAST, with cutoff values for significance at E ≤ 10^-8^, query and subject coverage of at least 40% and percent amino acid identity of at least 25%. For each such hit, all GO terms associated with the database sequence were transferred to a given fungal gene. In sum, of 125, 902 genes that were queried against the GO database, a total of 75, 511 were associated with GO terms.

### Functional distribution of gene duplicates

We compared the number of gene duplicates across twenty-two higher-level GO terms. For each GO term, we performed a chi squared test of the null hypothesis that the proportion of gene duplicates associated with that term was the same for both pathogens and non-pathogens [82,96]. We also compared the number of gene duplicates associated with individual GO terms in the Generic GO Slim ontology [97] in pathogenic *versus *non-pathogenic genomes. Here, as in the above, we tested whether the proportions of genes duplicates associated with a particular GO Slim term in pathogens *versus *non-pathogens were similar. To correct for the multiple hypothesis tests problems inherent to this approach, we judged results to be significant allowing a maximum false discovery rate (FDR) of 20% [96,98]. These two analyses were performed on all eleven genomes, on the nine euascomycete genomes and on the four pairs of genomes that showed differences in the overall distributions of gene duplicates (see above and Figure 2).

### Characterizing expansion and contraction of gene families

In the 500 largest gene families in our analyses, we tested for significant expansion or contraction in pathogenic genomes using a binomial test. Thus, for the global families, we calculated the proportion *p *(= 0.528) of all genes across all genomes in gene families of size two or greater that were observed in pathogenic species. This value was then used as the binomial parameter in the above test.

We also searched for significantly expanded families in the four instances where differences in gene family size distributions for pairs of genomes were evident (described above). To test for significance in these cases, a binomial test was again employed. For a given gene family, we compared the observed gene count for the lineage showing an overall greater magnitude of gene duplication (Figure 2) to the number genes contributed by the related species. In this context, the binomial parameter was *p *= 0.5. No attempt to account for differing genomes sizes was made in this analysis because the phylogenetic control makes it clear that any differences in genome size necessarily arose after the species pair in question shared a common ancestor.

### GenBank annotation for expanded and contracted gene families

We also performed BLAST searches for all expanded and contracted gene families against the GenBank non-redundant (nr) database [99]. Only sequences with BLAST E-values less than 10^-8^, which had at least 25% identity between subject and query, where the local alignment spanned at least 40% of the query sequence, and that were not annotated as "hypothetical protein" or "predicted protein" were retained.

## Abbreviations

Lineage-specific gene family expansion (LSE), Gene Ontology (GO), Repeat-induced point mutation (RIP), ATP-binding cassette (ABC), Major Facilitator Superfamily (MFS), GenomeHistory (GH), Single linkage clustering (SLC), Non-redundant (nr)

## Authors' contributions

AJP, IC and RAD conceived the study. AJP and DEB performed the analyses. GCC assisted with computational and statistical analyses. AJP and GCC wrote the manuscript. All authors read and approved the final manuscript.

## Supplementary Material

Additional file 1 Comparison of power law and exponential distributions for gene family sizes. These plots show that a power law provides a better fit for the distribution of gene family sizes in a genome than an exponential distribution. Click here for file

Additional file 2 Gene family sizes and functional annotation for gene families showing significant expansion in pathogens. This table summarizes gene family sizes and functional annotation for gene families that are expanded in fungal pathogens. Click here for file

Additional file 3 Gene family sizes and functional annotation for gene families showing significant contraction in pathogens. This table summarizes gene family sizes and functional annotation for gene families that are contracted in fungal pathogens. Click here for file

Additional file 4 Gene family sizes and functional annotation for gene families showing significant expansion in one member of a species pair. This table gives gene family sizes and functional annotation for gene families that are expanded in one member a species pair. Click here for file
