# Supplementary figures and images for: Altered patterns of gene duplication and differential gene gain and loss in fungal pathogens
Source: BMC Genomics. 2008 Mar 28;9:147. doi: 10.1186/1471-2164-9-147 (PMC2330156; doi:10.1186/1471-2164-9-147)

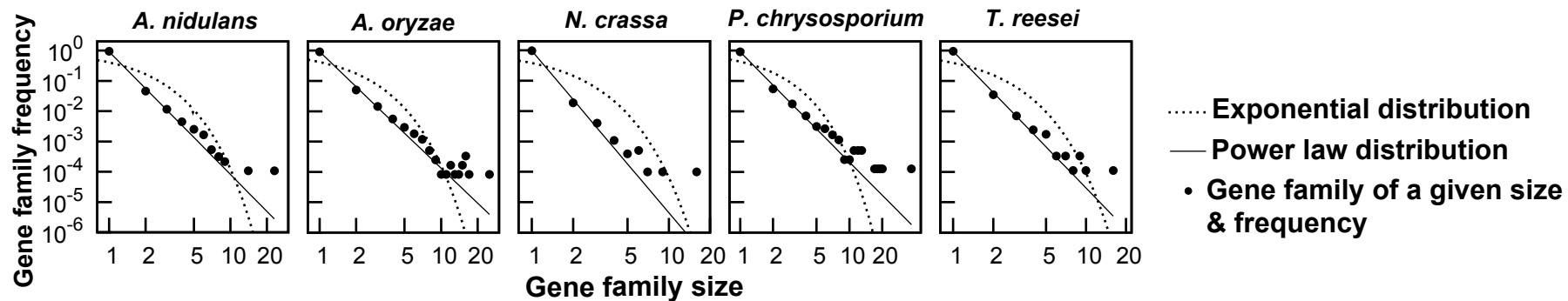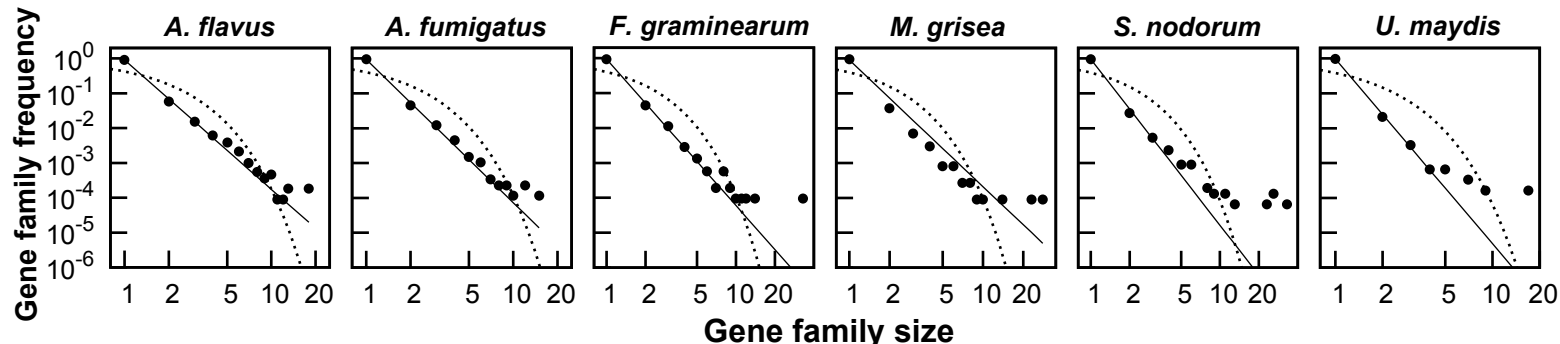

Supplement: Additional file 1 — Comparison of power law and exponential distributions for gene family sizes. These plots show that a power law provides a better fit for the distribution of gene family sizes in a genome than an exponential distribution. [file 1471-2164-9-147-S1.pdf]
