# Supplementary material for: Altered patterns of gene duplication and differential gene gain and loss in fungal pathogens
Source: BMC Genomics. 2008 Mar 28;9:147. doi: 10.1186/1471-2164-9-147 (PMC2330156; doi:10.1186/1471-2164-9-147)
Supplement: Additional file 2 — Gene family sizes and functional annotation for gene families showing significant expansion in pathogens. This table summarizes gene family sizes and functional annotation for gene families that are expanded in fungal pathogens. [file 1471-2164-9-147-S2.doc]

| ***Gene family size*** | ***Gene Ontology (GO)^a^ and GenBank^b^ functional annotation*** |
| --- | --- |
| 16 | receptor activity, ribonucleoprotein complex, ribosome, structural constituent of ribosome, protein biosynthesis, intracellular^a^; integral membrane protein, L-fucose permease^b^ |
| 16 | cell adhesion, galactose oxidase activity, copper ion binding, metal ion binding, oxidoreductase activity^a^; probable galactose oxidase precursor^b^ |
| 17 | hydrolase activity, acetylxylan esterase activity, pectin catabolism, cell wall catabolism, serine esterase activity, xylan catabolism, feruloyl esterase activity, extracellular region^a^; Feruloyl esterase, Tannase^b^ |
| 21 | integral to membrane, cell communication, integrin-mediated signaling pathway^a^; Regulatory P domain of the subtilisin-like proprotein convertases and other protease, other protease HAD superfamily hydrolase^b^ |
| 29 | hydrolase activity, aminopeptidase activity, peptidase activity, proteolysis^a^; aminopeptidase Y, vacuolar hypothetical protein^b^ |
| 32 | carbohydrate metabolism, hydrolase activity, acting on carbon-nitrogen (but not peptide) bonds, chitin binding^a^; polysaccharide deacetylase family protein, chitin deacetylase^b^ |
| 30 | membrane, amino acid-polyamine transporter activity, amino acid transport^a^; neutral amino acid permease^b^ |
| 37 | extracellular region, cutinase activity, metabolism, serine esterase activity, catalytic activity, hydrolase activity^a^; cutinase, cutinase precursor (Cutin hydrolase)^b^ |
| 40 | oxidoreductase activity, metabolism^a^; tyrosinase, monooxygenase^b^ |
| 109 | carbohydrate metabolism, hydrolase activity, acting on glycosyl bonds, cellulase activity, cellulose binding, hydrolyzing O-glycosyl compounds, extracellular region, nucleic acid binding, cellulose catabolism, polysaccharide catabolism, oxidoreductase activity, copper ion binding, metal ion binding, electron transport^a^; endoglucanase^b^ |
| 25 | integral to membrane^a^; C4-dicarboxylate transporter/malic acid transport protein^b^ |
| 40 | integral to membrane, receptor activity, metabolism, oxidoreductase activity^a^; transmembrane receptor, putative^b^ |
| 43 | pathogenesis, proteolysis, subtilase activity, negative regulation of enzyme activity, identical protein binding, peptidase activity, hydrolase activity, serine-type endopeptidase activity, vacuole^a^; cellular serine proteinase^b^ |
| 73 | membrane, transporter activity, integral to membrane, transport, carbohydrate transport, sugar porter activity, binding^a^; hexose transporter protein^b^ |
| 78 | transporter activity, integral to membrane, transport, membrane^a^; MSF transporter^b^ |
| 84 | electron transport, electron transporter activity, iron ion binding, integral to membrane, transport, transporter activity, membrane, carbohydrate transport, sugar porter activity^a^; maltose permease^b^ |

***Additional file 2: Gene family sizes and functional annotation for gene families showing significant expansion in pathogens***
