# Supplementary material for: Altered patterns of gene duplication and differential gene gain and loss in fungal pathogens
Source: BMC Genomics. 2008 Mar 28;9:147. doi: 10.1186/1471-2164-9-147 (PMC2330156; doi:10.1186/1471-2164-9-147)
Supplement: Additional file 3 — Gene family sizes and functional annotation for gene families showing significant contraction in pathogens. This table summarizes gene family sizes and functional annotation for gene families that are contracted in fungal pathogens. [file 1471-2164-9-147-S3.doc]

| ***Gene family size*** | ***Gene Ontology (GO)^a^ and GenBank^b^ functional annotation*** |
| --- | --- |
| 22 | integral to membrane, nucleotide binding, ATPase activity, ATP binding, nucleoside-triphosphatase activity, transport, membrane, coupled to transmembrane movement of substances, potassium ion transport, receptor activity, sulfonylurea receptor activity^a^; ABC multidrug transporter^b^ |
| 61 | heme binding, electron transport, monooxygenase^a^; *ordA*, oxidoreductase/cytochrome P450 monooxygenase, O-methylsterigmatocystin oxidoreductase (OMST oxidoreductase) (Cytochrome P450 64)^b^ |
| 27 | hydrolase activity, scytalidopepsin B activity, peptidase activity, aspartic-type endopeptidase activity, proteolysis, peptidase^a^; aspergillopepsin, acid glutamyl proteinase^b^ |
| 28 | oxidoreductase activity, acting on CH-OH group of donors, electron transport, FAD binding, alcohol metabolism^a^; glucose oxidase precursor, aryl-alcohol dehydrogenase^b^ |
| 68 | membrane, proton-transporting two-sector ATPase complex, hydrogen-transporting ATP synthase activity, rotational mechanism, membrane, integral to membrane, hydrogen-transporting ATP synthase activity, rotational mechanism, ATP synthesis coupled proton transport, integral to membrane, defense response^a^; vacuolar ATP synthase 16 kDa proteolipid subunit^b^ |

***Additional file 3: Gene family sizes and functional annotation for gene families showing significant contraction in pathogens***
