# Supplementary material for: Altered patterns of gene duplication and differential gene gain and loss in fungal pathogens
Source: BMC Genomics. 2008 Mar 28;9:147. doi: 10.1186/1471-2164-9-147 (PMC2330156; doi:10.1186/1471-2164-9-147)
Supplement: Additional file 4 — Gene family sizes and functional annotation for gene families showing significant expansion in one member of a species pair. This table gives gene family sizes and functional annotation for gene families that are expanded in one member a species pair. [file 1471-2164-9-147-S4.doc]

| ***Gene Family Size***  ***Additional file 4: Family sizes and functional annotation for gene families showing significant expansion in one member of a species pair*** | ***Species pair*** | ***Gene Ontology (GO)^a^ and GenBank^b^ functional annotation*** |
| --- | --- | --- |
| 19 | *F. graminearum*[[1]](#footnote-2)-T. reesei* | binding, membrane, transporter activity, integral to membrane transport, carbohydrate transport, sugar porter activity^a^; hexose transporter protein^b^ |
| 14 | *F. graminearum*-T. reesei* | integral to membrane, transport, membrane, transporter activity^a^; MFS transporter^b^ |
| 12 | *F. graminearum*-T. reesei* | hydrolase activity, hydrolyzing O-glycosyl compounds, polysaccharide catabolism, electron transport, cellulase activity, metal ion binding, acting on glycosyl bonds, extracellular region, oxidoreductase activity, carbohydrate metabolism, cellulose binding, cellulase activity, nucleic acid binding, copper ion binding^a^; endoglucanase^b^ |
| 47 | *F. graminearum*-T. reesei* | transferase activity, methyltransferase activity, S-adenosylmethionine-dependent methyltransferase activity^a^; methyltransferase *laeA*^b^ |
| 20 | *M. grisea*-N. crassa* | metal ion binding, electron transport, monooxygenase activity, heme binding, oxidoreductase activity, iron ion binding, acting on paired donors, with incorporation or reduction of molecular oxygen^a^; cytochrome P450 monooxygenase, othecene C-15 hydroxylase, trichothecene C-15 hydroxylase^b^ |
| 8 | *M. grisea*-N. crassa* | monooxygenase activity, iron ion binding, heme binding, electron transport, metal ion binding, oxidoreductase activity^a^; cytochrome P450 monooxygenase, gibberillic acid (GA) 20 oxidase^b^ |
| 12 | *M. grisea*-N. crassa* | iron ion binding, acting on paired donors, with incorporation or reduction of molecular oxygen, reduced flavin or flavoprotein as one donor, and incorporation of one atom of oxygen, electron transport, monooxygenase activity, heme binding, oxidoreductase activity, metal ion binding^a^; cytochrome P-450, alkane monooxygenase P-450^b^ |
| 14 | *M. grisea*-N. crassa* | transporter activity, integral to membrane, transport, membrane^a^; MFS transporter^b^ |
| 23 | *M. grisea*-N. crassa* | oxidoreductase activity, metabolism, hydrolase activity, glucose 1-dehydrogenase activity, membrane, antibiotic biosynthesis, melanin biosynthesis from tyrosine, aromatic compound catabolism, tetrahydroxynaphthalene reductase activity^a^; 1,3,8-trihydroxynaphthalene reductase^b^ |
| 12 | *A. nidulans**-*S. nodorum* | defense response, defense response to pathogen, binding, nucleoside metabolism, catalytic activity, methionine salvage, methylthioadenosine, nucleoside catabolism, adenosylhomocysteine nucleosidase activity, hydrogen-transporting ATPase activity, rotational mechanism, proton-transporting two-sector ATPase complex, membrane, hydrogen-transporting ATP synthase activity, integral to membrane, ATP synthesis coupled proton transport^a^; vacuolar ATP synthase 16 kDa proteolipid subunit, phosphorylase ^b^ |
| 18 | *A. nidulans**-*S. nodorum* | ATP binding, nucleoside-triphosphatase activity, integral to membrane, transport, nucleotide binding, ATPase activity, coupled to transmembrane movement of substances, membrane^a^; ABC transporter protein^b^ |
| 11 | *P. chrysosporium**-*U. maydis* | oxidoreductase activity, zinc ion binding, NADPH:quinone reductase activity^a^; enoyl reductase, alcohol dehydrogenase, zinc-containing alcohol dehydrogenase, zinc-containing, putative NADPH:quinone reductase and related Zn-dependent oxidoreductases^b^ |
| 19 | *P. chrysosporium**-*U. maydis* | catalytic activity, oxidoreductase activity, aromatic compound metabolism, monooxygenase activity, metabolism, electron transport, metabolism^a^; glucose oxidase precursor, aryl-alcohol dehydrogenase, Glucose-methanol-choline oxidoreductase^b^ |
| 9 | *P. chrysosporium**-*U. maydis* | oxidoreductase activity, acting on CH-OH group of donors^a^; aldo/keto reductase^b^ |
| 9 | *P. chrysosporium**-*U. maydis* | tripeptide aminopeptidase activity, aminopeptidase activity, hydrolase activity, subtilase activity^a^; tripeptidyl-peptidase, protease^b^ |
| 8 | *P. chrysosporium**-*U. maydis* | regulation of nitrogen utilization, transcriptional repressor activity, metabolism, oxidoreductase activity, response to stress, nucleus, D-lactaldehyde dehydrogenase activity, cytoplasm, NAD binding, catalytic activity, nucleotide-sugar, nucleotide-sugar metabolism^a^; putative oxidoreductase^b^ |
| 15 | *P. chrysosporium**-*U. maydis* | peptidase activity, hydrolase activity, autophagic cell death, pepsin A activity, proteolysis, aspartic-type endopeptidase activity^a^; aspartic protease precursor, protease^b^ |
| 25 | *P. chrysosporium**-*U. maydis* | carbohydrate metabolism, nucleus, glycerol dehydrogenase (NADP+) activity, cell, D-xylose catabolism, arabinose catabolism, 2,5-didehydrogluconate reductase activity, oxidoreductase activity, response to salt stress, cell, cytoplasm, alcohol dehydrogenase (NADP+) activity, D-xylose metabolism, aldo-keto reductase activity^a^; aldo-keto reductase, 2,5-didehydrogluconate reductase^b^ |
| 14 | *P. chrysosporium**-*U. maydis* | cell wall (*sensu* Fungi), hydrolase activity, acting on glycosyl bonds, carbohydrate metabolism, hydrolyzing O-glycosyl compounds^a^; endo-1,3(4)-beta-glucanase^b^ |

1. “*” denotes which member of a species pair showed larger gene families [↑](#footnote-ref-2)
